# Monitoring microvascular changes over time with a repositionable 3D ultrasonic capacitive micromachined row-column sensor

**DOI:** 10.1126/sciadv.adr6449

**Published:** 2025-03-26

**Authors:** Cyprien Blanquart, Léa Davenet, Julien Claisse, Mallorie Giroud, Audren Boulmé, Edgard Jeanne, Mickaël Tanter, Mafalda Correia, Thomas Deffieux

**Affiliations:** ^1^ Physics for Medicine Paris, Inserm U1273, ESPCI Paris, PSL University, CNRS UMR 8063, Paris, France.; ^2^ MODULEUS, Tours, France.

## Abstract

eHealth devices, including smartwatches and smart scales, have the potential to transform health care by enabling continuous, real-time monitoring of vital signs over extended periods. Existing technologies, however, lack comprehensive monitoring of the microvascular network, which is linked to conditions such as diabetes, hypertension, and small vessel diseases. This study introduces an ultrasound approach using a capacitive micromachined ultrasound transducer row-column array for continuous, ultrasensitive three-dimensional (3D) Doppler imaging of microvascular changes such as hemodynamic variations or vascular remodeling. In vitro tests and in vivo studies with healthy volunteers demonstrated the sensor’s ability to image the 3D microvascular network at high resolution over different timescales with automatic registration and to detect microvascular changes with high sensitivity. Integrating this technology into wearable devices could, one day, enhance understanding, monitoring, and possibly early detection of microvascular-related health conditions.

## Teaser

3D ultrasound Doppler imaging sensor allows long-term microvascular monitoring.

## INTRODUCTION

In recent years, eHealth devices such as smartwatches or smart scales have demonstrated tremendous potential in transforming health care through continuous and real-time monitoring of vital signs. Modern wearables combine various physical measurement techniques to collect health data, including accelerometers and gyroscopes for activity tracking or fall detection, photoplethysmography (PPG) for heart rate monitoring, electrocardiograms (ECGs), or bioimpedance sensors for body composition analysis (*1*). ECG sensors, for example, measure the electrical activity of the heart to detect irregular heart rhythms such as atrial fibrillation, allowing users to take spot readings and export ECG graphs for medical consultations (*2*). Bioimpedance sensors in smart scales can provide detailed body composition analysis, including measurements of body fat, muscle mass, and visceral fat in addition to conventional weight measurements. Newer models of scales have capabilities such as measuring pulse wave velocity (PWV) (*3*), which provides a noninvasive and real-time assessment of arterial stiffness, a key biomarker of cardiovascular health. In any case, monitoring is especially valuable because it allows for the comparison of readings longitudinally over months and potentially years, providing access to long-term trends in a specific person or larger population. Despite the rapid rise of eHealth technologies, available sensor capabilities remain limited and are decades behind what can be measured in a clinical setting by full-fledged medical imaging devices such as ultrasound, computed tomography, or magnetic resonance imaging scanners with trained operators.

In this work, we focus on the monitoring of the micro vascularization, e.g., the intricate network of small blood vessels, which is linked to various medical conditions, including diabetes mellitus (DM) (*4*), hypertension (*5*), atherosclerosis (*6*), chronic kidney disease (*7*), rheumatoid arthritis (*8*), and connective tissue disorders such as scleroderma (*9*). Changes in microvascular health are also critical indicators in inflammation and healing processes, particularly in monitoring grafts and wounds, where adequate blood supply through these microvessels is essential for successful healing and integration of the grafted tissue (*10*). Similarly, assessing limb perfusion in patients with vascular pathologies is crucial for evaluating function. This is particularly important for patients with DM, who are at elevated risk for vascular complications such as generalized vascular lesions and foot ulcers (*11*) and represent a major public health challenge with half a billion people affected worldwide (*12*). Systemic sclerosis, a rare chronic autoimmune disease characterized by connective tissue fibrosis, also leads to vascular abnormalities (*13*). The ability to continuously monitor microvascular health could thus enhance the early detection and treatment of these conditions.

In the extremities, the vascular system is dense and forms a branched network. For example, in the hand the vessels’ diameter goes from 2 to 3 mm in the wrist arteries to 1 to 2 mm in the finger. Those vessels then supply the subcutaneous plexus, composed of 100- to 500-μm vessels and located between 1 and 5 mm below the surface. Smaller vessels are present in the dermis plexus (*14*). Resolution of a few hundred micrometers is thus mandatory to image the subcutaneous plexus. Existing imaging techniques of microvasculature, such as optical coherence tomography (OCT), laser Doppler flowmetry (LDF), and photoacoustic (PA) imaging, provide valuable insights into superficial microvessels with impressive spatial resolutions down to 10 μm (*15*). However, integrating these methods into small, affordable, and robust wearable devices is especially challenging. OCT requires complex hardware setups and optical calibration (*16*), LDF is sensitive to tissue optical properties (*17*), and PA involves either bulky equipment (*18*) or low penetration depth (*19*, *20*). These limitations hinder their practicality for embedded use and continuous, real-time monitoring of the microvasculature.

Ultrasound technology, particularly Doppler ultrasound, can address these limitations (Table 1). Over the past decade, advancements in electronics miniaturization, soft materials, and flexible electronics have spurred the development of wearable ultrasound devices for various biomedical applications. Recently, stretchable ultrasound probes were introduced (*21*–*24*), highlighting the potential of wearable ultrasound devices to expand beyond traditional applications. However, despite these advancements, current wearable ultrasound methods have not yet achieved the capability to provide detailed and continuous three-dimensional (3D) imaging of microvessels. Their primary focus has remained on imaging larger structures (*25*, *26*) or large vessel blood flows (*27*–*29*). Recently, a team achieved 3D imaging of the Willis circle through the skull and could extract spectral Doppler of millimetric vessels (*30*).

**Table 1.** Comparison of different imaging techniques.

	Type	Penetration depth	Resolution	Field of view	Applications
**Optical coherence tomography angiography**	Optical	1–2 mm	~10 μm	Superficial 3D with scan	Superficial microvessels at very high resolution in skin or retina
**Laser Doppler flowmetry**	Optical	1 mm	~100–500 μm	Superficial 3D with scan	Monitoring blood flow on skin
**Photoacoustic imaging**	Optical, ultrasound	1–15 mm	~50–100 μm depth dependent	3D with scan	Imaging microvessels
**Clinical Doppler ultrasound**	Ultrasound	5–80 mm	~200–500 μm	Deep 2D	Imaging larger vessels like arteries and veins
**3D RCA Doppler ultrasound**	Ultrasound	1–15 mm	~ 100 μm	3D imaging	Imaging of small blood flow networks with blood volume

Traditional ultrasound platforms are also typically restricted to 2D imaging and have limited sensitivity to the small blood flows present in microvessels. Meanwhile, the development of ultrafast ultrasound imaging has revolutionized the field by enabling high–frame-rate acquisitions capable of capturing 10,000 images per second, compared to the conventional rate of 50 images per second (*31*). Ultrafast ultrasound imaging has been effectively used for Doppler ultrasonography, where it substantially boosts sensitivity to small blood flows, making it possible to detect and measure even the slowest blood flow rates that traditional ultrasound methods might miss (*32*). One of the applications of ultrafast ultrasound imaging also lies in the measurement of PWV in peripheral arteries (*33*). As discussed earlier, PWV is an interesting parameter linked to arterial stiffness, an important biomarker for cardiovascular health.

However, ultrasound imaging is known to be operator dependent, due to the difficulty in positioning the probe reliably between exams, and, as such, its use as a smart sensor with longitudinal follow-up would mandate a true volumetric acquisition. However, the shift from 2D to 3D ultrafast ultrasound imaging requires advanced technology that is not even available to the most advanced clinical scanners. This technology relies on 2D matrix probes of *N* × *N* elements and scanners with thousands of channels, enabling 3D imaging rates that exceed 1000 volumes per second. This complexity and cost significantly limit their use to specialized research environments and hinder widespread adoption. To overcome these challenges and expand the use of 3D ultrasound imaging into the smart sensor context, reducing the number of electronic channels is crucial. Different methods have been implemented to tackle this issue, including large element arrays coupled with encoding masks (*34*) or scans of linear arrays (*35*). The row-column array (RCA) approach also offers a promising solution by reducing the number of channels from *N* × *N* to *N* + *N* (*36*). This can be achieved by addressing each element through row and column controls rather than individual connections. This approach provides a simple, flexible, and cost-effective alternative to fully populated 2D arrays. By leveraging the RCA configuration, it is possible to perform sensitive 3D Doppler imaging while significantly reducing the hardware complexity and cost. This kind of sensor was successfully made and used for ultrasound imaging on phantoms (*37*), on large blood vessels (*38*), and on microvessels with enough sensitivity to detect small hemodynamic changes due to the neurovascular coupling effect in rodents (*39*). More recently, this approach has been used for microvascular imaging with ultrasound localization microscopy, a method based on the injection of microbubbles (*40*), and for submandibular glands blood flow imaging (*41*).

The RCA architecture fits well with capacitive micromachined ultrasound transducers (CMUTs) and has been successfully implemented in the past (*42*–*44*). CMUT provides a simplified fabrication and integration process by leveraging advanced microfabrication techniques. This implies lower mass production cost on a scale, which is crucial for smart sensor development. Those studies also focused on phantoms (*45*) and large vessels imaging (*46*), but, to our knowledge, no microvessel imaging has been described with these technologies yet. The bias tension applied during these studies can be rather high, around 100 V, which could also limit its use, especially for portable or wearable devices.

Our study proposes a contrast agent free microvascular monitoring method with a repositionable 3D ultrasound RCA sensor based on low-tension CMUT technology and ultrafast imaging and built around an evaluation research platform and sensor that is not yet a fully wearable device.

In the first part of this study, we design and investigate the electromechanical properties of the CMUT RCA sensor, including impedance measurements and acoustic performance characterization using a hydrophone and pulse-echo testing. We implement ultrafast acoustic sequences, enabling 3D imaging and optimized for low acoustic power. We compare different imaging sequences in vitro, such as orthogonal plane-wave (OPW) imaging, single-array plane-wave (SPW) imaging, synthetic aperture (SA) imaging, and Hadamard-encoded synthetic aperture (HSA) imaging, to determine the optimal energy/performance point of the sensor for micro flow imaging.

We then investigate the sensor’s capability in assessing the microvasculature in various body parts, including fingers, wrists, and toes in 3D Doppler in vivo studies. We evaluate the 3D registration of micro-Doppler volumes over various timescales for longitudinal measurements and the sensitivity of the sensor to blood volume changes in response to contralateral temperature stimuli.

Last, we explore the ability of the sensor to detect the mechanical pulse wave (MPW) propagating in the tissue around peripheral arteries. We evaluate the accuracy of this for cardiovascular assessment including heartbeat and mean PWV and compare it to other methods.

The approach provides a repositionable operator-independent solution potentially compatible with wearable form factors such as watches, scales, and armbands. On the basis of our findings, we anticipate that a fully integrated and miniaturized platform, similar to the Butterfly iQ platforms (Butterfly Network Inc., Burlington, MA, USA), and, on the basis of integration of CMUT (*47*) with custom ASIC (application-specific integrated circuit), could be developed to capitalize on these capabilities and enable microvascular health monitoring in various wearable device formats.

## RESULTS

### Design and working principle of the CMUT RCA

**Fig. 1.**
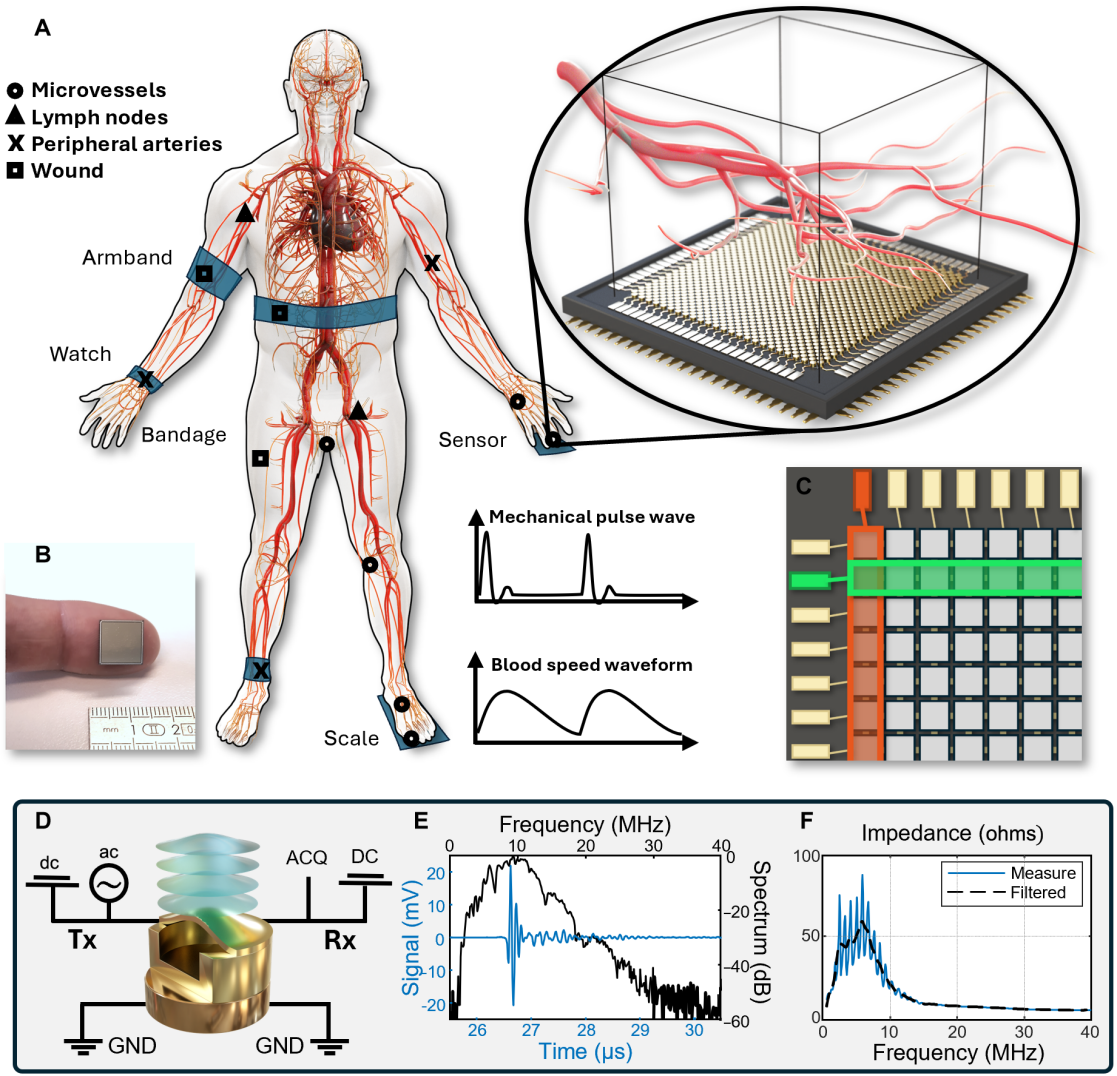
Design and working principle of the CMUT RCA. (**A**) Schematic showing the CMUT row column addressed array sensor and its ability to perform Doppler imaging of the superficial peripheral micro vessels and detect pulse waves. Different body parts could be imaged for blood flow monitoring including finger, palm, wrist, foot, superficial lymph nodes, or wounds. The sensor can also detect pulse waves in the tissue and the blood vessels. (**B**) Pictures of the 9.6 mm–by–9.6 mm sensor on an index finger for size comparison. (**C**) Display of the electrodes addressing the elements by row and column. One row (green) and one column (orange) are highlighted. The information of the 8192 transducers is gathered in 64+64 channels. (**D**) Schematic of a CMUT cell with its electric circuit for transmit (Tx) and receive (Rx) events. The CMUT is biased by dc and excited by ac tensions. The radio-frequency signal is measured by an acquisition platform (ACQ). (**E**) Pulse-echo signal of element 34 and its spectrum for an impulse transmit. (**F**) Real part of the measured electric impedance of the element 34 filtered to remove oscillations.

A conceptual schematic of the CMUT RCA is shown on Fig. 1A. The sensor detects internal structures of the body through ultrasound pulse-echo imaging. The ultrasound array fulfills the requirements for qualitative B-mode and Doppler imaging with a 9.6 mm–by–9.6 mm field of view (FOV). Temporal resolution is also good enough to detect cardiac variations and pulse waves in the blood and in the tissue. A picture of the actual RCA CMUT chip on a finger is shown for comparison on Fig. 1B for size comparison. This area is large enough to detect superficial peripheral microvessels but small enough to adapt to skin curvature, and we successfully used it for different body areas including fingers, palm, wrist, and foot.

The CMUT cells are addressed by lines and columns as presented in Fig. 1C. This electrode pattern reduces both the chip complexity and the hardware required for the pulse echo imaging and processing. After the dicing of the individual chips, they were tested and could handle both 35-V dc and ±15-V ac tension with no electrical or mechanical defects for both transmission and reception mode (Fig. 1D).

To further evaluate the electromechanical properties of the sensor, we measured the impedance of the lines and rows. We obtained an average of 58.5 ± 1.7 ohms at 5.81 ± 0.09 MHz over the elements. We also characterized the acoustic performances of the sensor with a hydrophone and with pulse echo on an interface. The mean center frequency of the array is 9.02 ± 0.19 MHz, with a 73 ± 5% bandwidth (−6 dB). The results of the 34th element, a representative of the array, are plotted for pulse echo signal (Fig. 1E) and for electrical impedance (Fig. 1F).

### Optimization of the ultrasound sequence for microvascular imaging

**Fig. 2.**
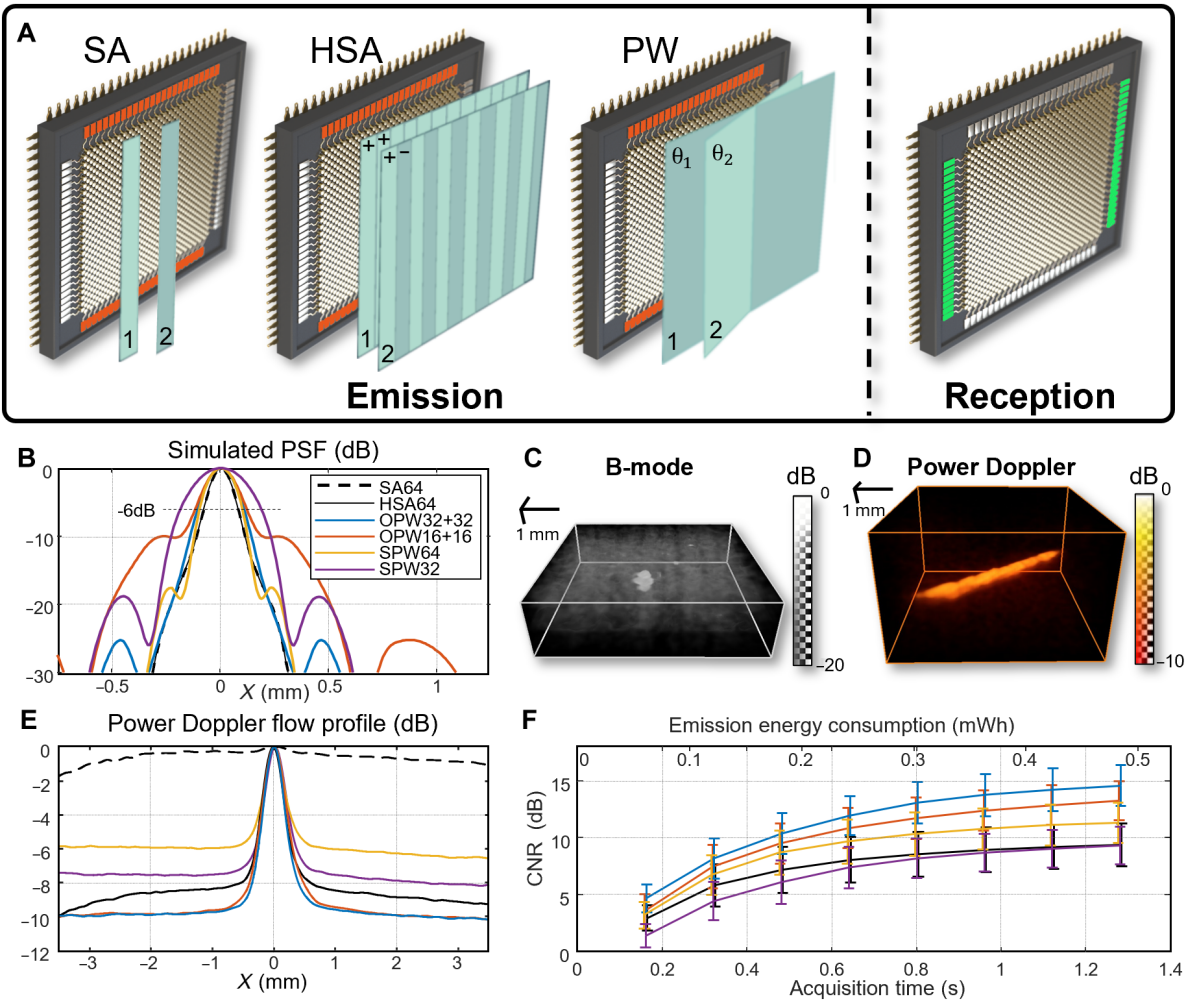
Optimization of the ultrasound sequence for microvascular imaging. (**A**) Schematic of the different emission methods: single-element synthetic aperture (SA), Hadamard-encoded synthetic aperture (HSA), plane waves (PW). For each transmit on an array’s channels (orange), and the other array is used for reception (green). This cross emission/reception enables 3D focusing with the RCA. Only one array emits for SA, HSA, and single-array plane waves (SPW), while both emit sequentially for orthogonal plane waves (OPW). (**B**) Simulated point spread function (PSF) profiles for the different imaging methods and for different numbers of plane waves. Full width at half maximum (FWHM) and lobes level are used as a metric for image quality. (**C**) B-mode of 180-μm diameter bead and (**D**) power Doppler of blood-mimicking fluid flowing in an agar/gelatin phantom (1.28 s of OPW32+32 sequence). (**E**) Profiles of the flow in the phantom for FWHM and contrast-to-noise ratio (CNR) measurement. (**F**) Comparison of the phantom power Doppler CNR for the different imaging methods, acquisition time, and power consumption for emission.

We compared different imaging methods with our sensor to get the best trade-off between resolution, sensitivity, and computational complexity for micro blood vessels imaging. Plane-wave sequences have a good sensitivity but can be limited with their resolution depending on the angular opening (*39*). Single-element synthetic apertures are known for their resolution but can be limited by their sensitivity (*48*). Hadamard encoding of these subapertures can improve the signal-to-noise ratio (SNR) and, thus, the sensitivity (*49*). The different sequences tested for this study: SA, HSA, OPW, and, lastly, SPW imaging are displayed in Fig. 2A. For each sequence, we cross the emission and reception array for 3D focusing with the RCA.

To characterize each imaging sequence, we first used ultrasound propagation simulation in a medium with a single scatterer. We computed squared point spread functions (PSFs) by beamforming the simulated echo signal to measure the full width at half maximum (FWHM; −3 dB) (Fig. 2B and fig. S1), which is a good estimate of the power Doppler resolution. The results are gathered in Table 2. SA and HSA got best results with a 193-μm FWHM. OPW32+32, OPW16+16, and OPW8+8 plane waves got 237, 279, and 291 μm, respectively. SPW64, SPW32, and SPW16 got 186, 334, and 692 μm, respectively, in the plane-wave direction.

**Table 2.** Results of the simulation of the different sequences.

	SA64	HSA64	OPW8+8	OPW16+16	OPW32+32	SPW16	SPW32	SPW64
**FWHM, −3 dB (μm)**	193	193	291	279	237	692	334	186
**Side lobes (dB)**	−50 dB	−50 dB	−7	−10	−26	−18	−19	−47

The side lobes maximum level was also measured, and SA and HSA were also the best option with −50 dB against OPW [−26 dB (32+32), −10 dB (16+16), and −7 dB (8+8)] and SPW [−47 dB (64), −19 dB (32), and −18 dB (16)]. These results are also in Table 2. The plane-wave delta angle was set at 1° to avoid grating lobes due to plane-wave emission, and, thus, none was observed. Due to the sensor pitch length and the low imaging depth (4.8 mm), grating lobes are noticeable but below −30 dB.

We then performed 3D B-mode imaging and Doppler imaging on phantoms to confirm imaging abilities of the sensor in real conditions. The RCA CMUT could image in the 180-μm-diameter steel bead at 6-mm depth in the polyvinyl chloride (PVC) phantom. The OPW32+32 plane-wave sequence achieved a 490-μm FWHM and a −25-dB SNR (Fig. 2C). Power Doppler imaging performance was then characterized, thanks to a homemade gelatin/agar phantom with a 500-μm cavity at 10-mm depth. We obtained the 3D power Doppler volume by filtering the speckle due to static agar scatterers thanks to singular value decomposition (SVD). Only the blood-mimicking fluid in the pipe remained in the filtered volume (Fig. 2D). We compared the orthogonal profiles of the pipes (Fig. 2E). OPW32+32 sequence got the best FWHM (547 μm) against HSA (617 μm) and OPW16+16 (629 μm). SA could not detect the flow.

The contrast-to-noise ratio (CNR) was computed to compare the different methods (Fig. 2F). For each acquisition time OPW32+32 got better results, reaching 14.6 ± 1.8 dB for 1.28 s of acquisitions. The other sequences achieved 13.3 ± 1.7 dB (OPW16+16), 11.3 ± 1.7 dB (SPW64), 9.4 ± 1.9 dB (HSA64) and 9.3 ± 1.6 dB (SPW32). SA could not achieve a CNR high enough for proper imaging. A shorter OPW32+32 sequence of 0.64 s (200 frames) could also be used with high CNR 12.0 ± 1.7 dB. It was chosen for the following study. The B-mode imaging frame rate of this sequence is 312.5 Hz, while the power Doppler is up to 1.33 Hz.

Acoustic safety measurement was performed on the sequence OPW32+32 with the hydrophone. It shows a mechanical index (MI) of 0.23 (MI < 0.3: no limitation in any imaging scenario including contrast agent or ocular imaging), a soft tissue Thermal Index (TIS) of 1.28 (TIS < 1.5: no time limitation), and a spatial peak temporal average intensity (ISPTA) of 26.9 mW/cm^2^ (ISPTA < 720 mW/cm^2^).

Electronic circuit simulation showed an average power consumption of 1.36 W for the pulse emission during the acquisition. The lowest consuming sequence is, thus, SA because of its single-element transmit [0.49 megawatt-hour (mWh) for 1.28-s acquisition]. The energy consumed by the other sequences is 64 times higher (31.0 mWh for 1.28 s acquisition). The emission sequence consumptions are shown on Fig 2F. The lower SNR of SA could be explained by the lower energy emitted during the sequence. Hadamard encoding increases this CNR, but the lack of symmetry between positive and negative emission could explain the higher noise compared to plane-wave sequences. SPW, and, especially, SPW64, weak CNR could be explained by the directivity of the elements that reduce the energy of high-angle plane waves.

### In vivo Doppler microvascular imaging for a repositionable sensor

**Fig. 3.**
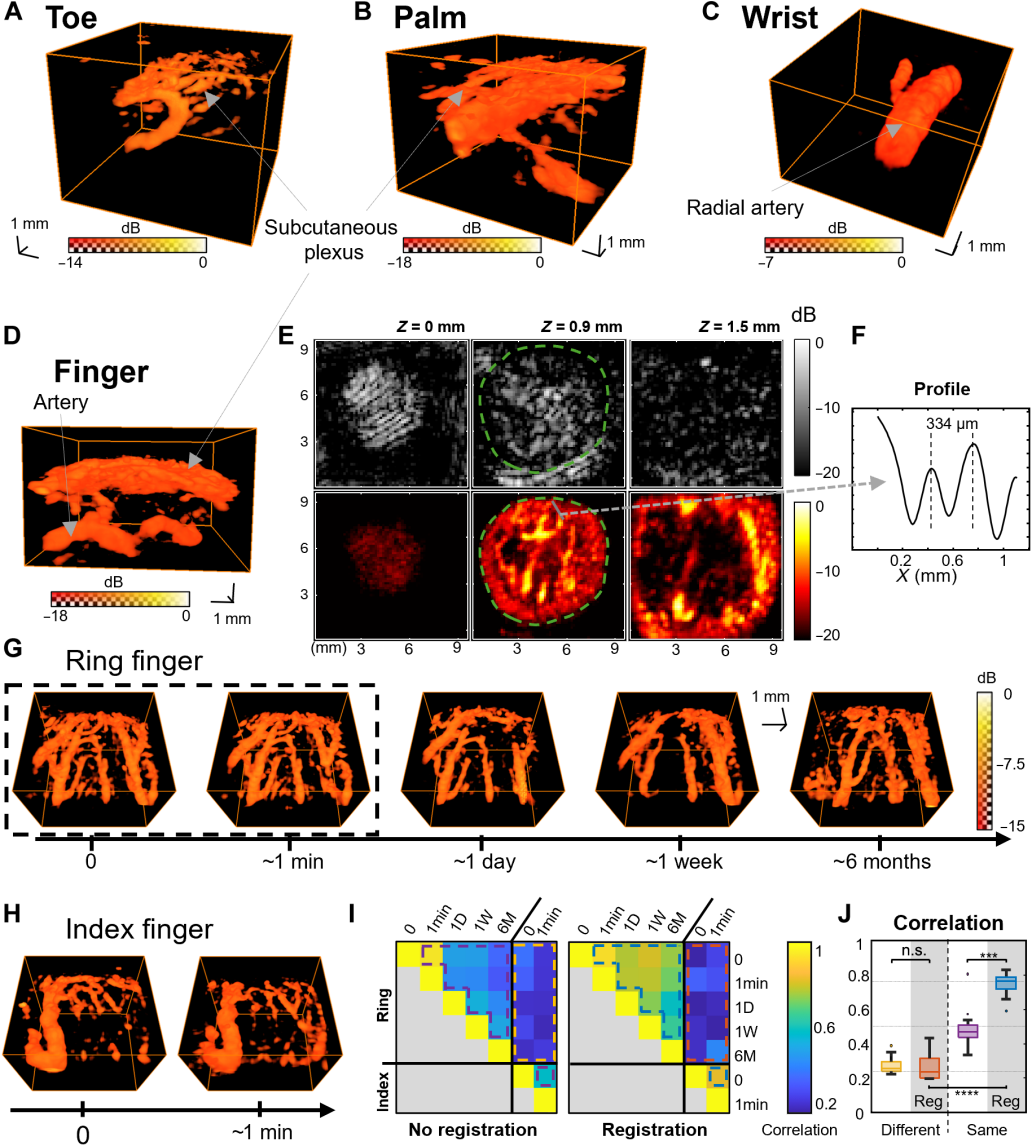
In vivo Doppler microvascular imaging for a repositionable sensor. (**A**) 3D power Doppler volume of a human big toe, (**B**) hand palm, (**C**) wrist, and (**D**) ring finger with the holders [sequence: 1.28 s of OPW32+32, pulse repetition frequency (PRF) of 20 kHz] (movies S1 and S2). (**E**) B-mode and power Doppler slices at 0-, 0.9-, and 1.5-mm depth in the finger (sequence: 8.5 s of OPW32+32, PRF of 3 kHz). The green line delineates the skin fingerprint and the blood vessels. (**F**) The profile of two thin and close blood vessels for resolution assessment. (**G**) Ring finger and (**H**) index finger imaged over 6 months for a longitudinal automatic registration study (sequence: 0.64 s of OPW32+32, PRF of 20 kHz). The first two acquisitions of the ring finger are made without removal from holder. (**I**) Correlation of the power Doppler volumes before and after registration. (**J**) Correlations of the power Doppler gathered for different cases: same or different fingers with and without registration. *P* values were computed with Student’s *t* test (***P < 0.001; ****P < 0.0001; n.s., not significant).

The CMUT RCA can address various body parts for superficial imaging. Big toe (Fig. 3A), palm (Fig. 3B), wrist (Fig. 3C), and fingers (Fig. 3D) were successfully imaged though 3D B-mode and power Doppler using the specific holder design.

In the human wrist (Fig. 3C), the volumes show the radial artery (2.5-mm diameter) and a junction with a palmar carpal branch (1-mm diameter). On the other side of the wrist, the ulnar artery (2.7-mm diameter) and vein (1.7-mm diameter) could also be imaged. They are located respectively at 3.9 and 5 mm under the skin and are alongside each other. Cephalic veins could also be imaged in the superficial layers.

In the palm (Fig. 3B), a subcutaneous plexus is also noticeable. In addition to the small and dense vessel network, bigger superficial vessels could be imaged. Vessels are also connected to deeper tissue layers, around the thumb muscles.

In the finger and the toe (Fig. 3, A and D), the dense subcutaneous plexus located around 1 mm under the surface was well detected by our sensor with vessels from 300 to 800 μm (Fig. 3E). Bigger arteries (1-mm diameter) are located at a bigger depth (4 mm) and supply the plexus with blood. In superficial layers, the subcutaneous plexus is directly connected to the dermal plexuses and, then, the capillary loops by vertical arteries and veins. The vessels here are too thin (less than 100 μm) and thus cannot be resolved by the sensor. However, global perfusion in the voxels of this area can still be measured with power Doppler imaging. Last, no blood vessels are in the epidermal layer. The B-mode of this layer shows fingerprints on the finger surface (Fig. 3E). Blood vessels are in different voxels than fingerprints and, thus, are not tissue artifacts. The phalanx bone is detected between 5.5- and 7.3-mm depth in the distal phalanx.

These in vivo measurements confirm the phantom study conclusions on the imaging method of a human finger (fig. S1, A to D). OPW32+32 got the best results with an SNR of 23.3 dB in comparison with 20.2 dB for OPW16+16, 18.5 for HSA, and 9.7 dB for SA.Thickness at −3 dB of a thin vessel was also measured; it showed comparable results for SA, HSA, and OPW32+32 (356, 360, and 353 μm) but not for OPW16+16 (456 μm). The chosen sequence OPW32+32 was also able to resolve vessels separated by 334 μm (Fig. 3F). We also compared XDoppler processing and OPW in vivo (fig. S2, C to F). A better SNR was achieved for both OPW16+16 and OPW32+32 acquisition (29 instead of 20.2 dB and 31 instead of 23.3 dB) and an improved resolution that shows papillae for OPW16+16. However, some vessels are not detected in the XDoppler imaging. So, for OPW32+32, the similar resolution and the increased computational complexity of the XDoppler confirm the choice of using simple coherent summation: OPW.

We performed a longitudinal 3D power Doppler study on a healthy volunteer several times for 6 months (Fig. 3G). The acquisitions were made on the ring finger with no other guidance than the acoustic window and a stop wall. Two acquisitions on the index finger were also added for volume comparison and to test the registration algorithm (Fig. 3H). Same finger automatic registration converged in 100% of the cases with a maximum displacement measured of 0.83 mm and a maximum rotation angle of 5.3°, leading to a minimum common volume of 84% of the sensor FOV. To assess the registration, we computed Pearson correlation between the power Doppler of each acquisition (Fig. 3I). Voluntary bigger translation and rotation have also been studies in (fig. S4).

The correlation significantly increased with the registration for the same finger (from 49.0 ± 13.7% to 74.9 ± 4.4%, *P* = 1.26 × 10^−4^, *n* = 10, paired *t* test) but not for different fingers (from 27.0 ± 5.4% to 26.4 ± 7.5%, *P* = 0.85, *n* = 10, paired *t* test) (Fig. 3J). The correlation was also significantly higher for the same finger than for different after registration (*P* = 3.94 × 10^−11^, *n* = 10, Welch test).

The highest correlation was obtained for the two first acquisitions separated by a minute and without removal of the finger out of the holder. In this case, registration was useless. Power Doppler volume changed over the 6 months, but we could not detect any temporal trend within our dataset.

### A pulse wave sensor for cardiovascular assessment

**Fig. 4.**
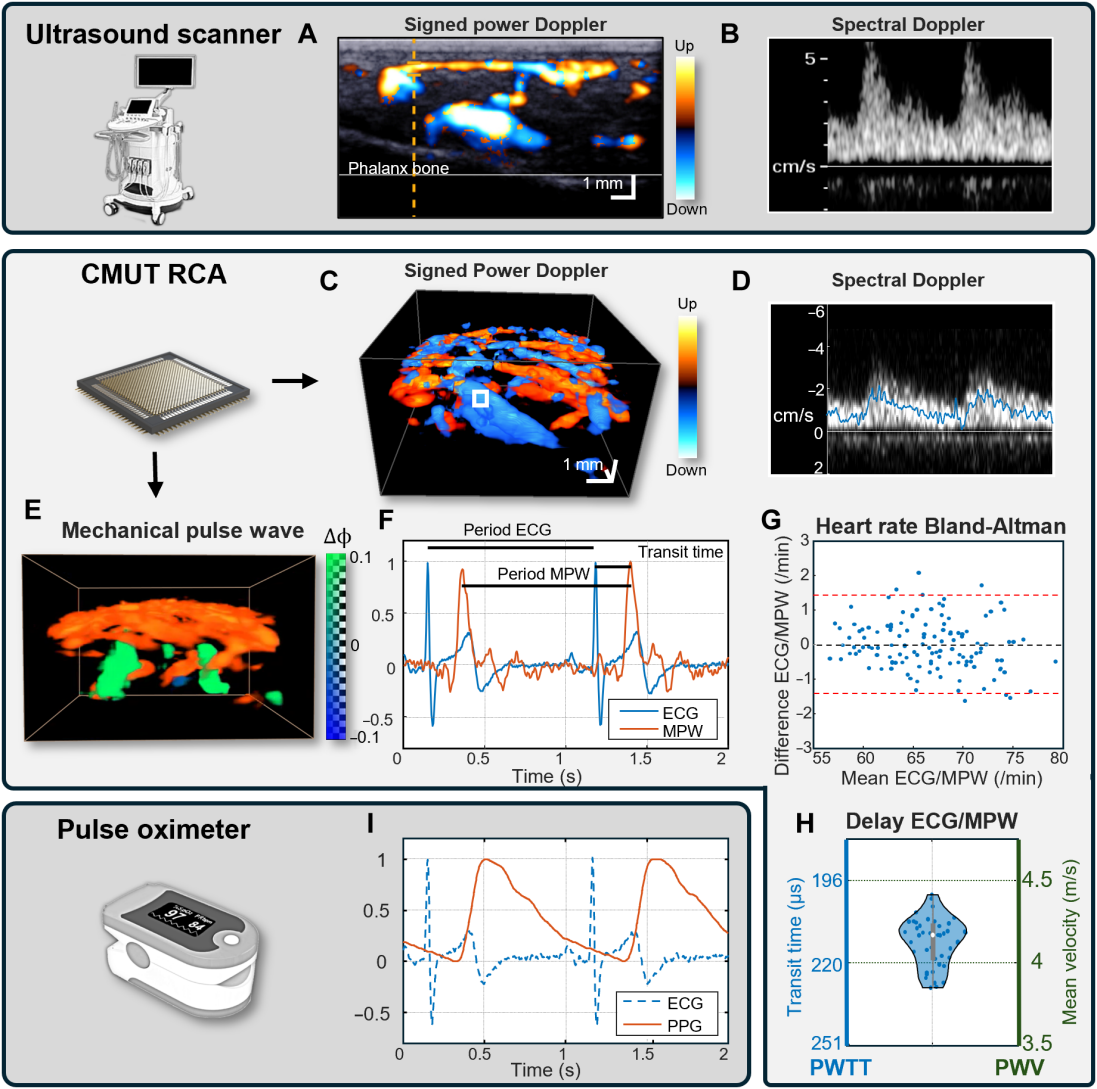
Systemic peripheral vasomotion monitoring. (**A**) 2D signed power Doppler and (**B**) spectral Doppler waveform of a distal phalanx of the ring finger acquired with a clinic ultrasound scanner and a 15-MHz linear array probe. (**C**) 3D signed power Doppler (sequence: OPW32+32, PRF of 20 kHz). (**D**) Spectral Doppler waveform of the same phalanx acquired with the CMUT RCA sensor for the white underlined region of interest (ROI) of the volume (sequence: OPW8+8, PRF of 20 kHz). (**E**) Periodic deformation from bottom to top (green) around the deeper artery of the acquisition (movie S3). (**F**) Ultrasound pulse wave detection synchronized with the ECG. The heart rate is measured between ECG R-peaks and between ultrasound deformation peaks. Delay between ECG and Ultrasound peaks is defined as the pulse wave transit time (PWTT). (**G**) Bland-Altman of the heart rate measured by ECG and Ultrasound. The bias is 0.03 beats per minute (BPM), and 95% confidence interval is ±1.7 BPM, for a mean value of 69.7 BPM. (**H**) Histogram of the PWTT, the mean value is 214 ms (±8 ms). PWV is obtained with a heart-finger distance of 88 cm, measured on the volunteer. The mean value is 4.11 m/s (±0.15 m/s). (**I**) Photoplethysmography (PPG) performed with an oximeter on the same phalanx for comparison.

To evaluate the ability of the sensor to detect flow direction in the finger subcutaneous plexus, we performed a signed power Doppler (Fig. 4C). Acquisitions performed with the sensor and the OPW32+32 sequence were similar to the 2D signed power Doppler slices acquired on a clinical ultrasound scanner (Aixplorer, SuperSonic Imagine, Aix-en-Provence, France) (Fig. 4A). We also computed the spectral Doppler to estimate blood flow axial speed waveforms during several cardiac cycles with the OPW8+8 sequence. No aliasing is detected for the vessels in the plexus with low axial velocity. The average spectral Doppler of a 450-μm cube region of interest (ROI) underlined in Fig. 4C is shown in Fig. 4D. The waveform is also similar to the spectral Doppler acquired with the clinical ultrasound scanner for plexus vessels (Fig. 4B). The resistive index (RI) could be computed on the average speed of the spectral Doppler. The RCA CMUT measured 0.57 ± 0.05 (*n* = 7 spectral Doppler cycle) in agreement with the measure on the clinic ultrasound RI = 0.55 ± 0.06 (*n* = 15 acquisitions on same finger plexus vessels). The RCA CMUT could not assess the waveform in bigger vessels due to aliasing and in horizontal vessels due to lack of Doppler signal.

To improve the pulse wave detection, we estimated the deformations in the finger by frame-to-frame lag autocorrelation. We observed an MPW around deeper arteries (Fig. 4E). Our method could extract the MPW from the volumetric phase shifts without motion artifacts. The signal is a peak when blood arrives in the finger, followed by attenuated oscillations. We compared this signal with the ECG measured simultaneously on a volunteer (Fig. 4F) and assessed the accuracy and precision of the CMUT RCA using the ECG as reference. The Bland-Altman plot of these methods (*n* = 125 cardiac cycles) (Fig. 4G) shows a mean difference of 0.03 beats per minute (BPM) for a mean value of 69.7 BPM. The 95% limits of agreement are ±1.7 BPM, which is lower than usual heart rate variability (HRV; ±4.9 BPM in the data used here).

The delay between the contraction of the ventricles (R-peak of the ECG) and the arrival of the MPW in the finger can be used as a measurement of the pulse wave transit time (PWTT) and PWV. We measured a delay of 214 ± 8 ms (Fig. 4H). This corresponds to a mean PWV of 4.11 m/s (±0.15 m/s) between the heart and the finger. We also performed photoplethysmography (PPG) of the finger with a commercial oximeter to compare the waveforms between both approaches (Fig. 4H). The peaks of the PPG were wider (FWHM of 410 ± 13 ms) than the pics of the MPW (FWHM of 65 ± 4 ms).

### Detection of hemodynamic changes

**Fig. 5.**
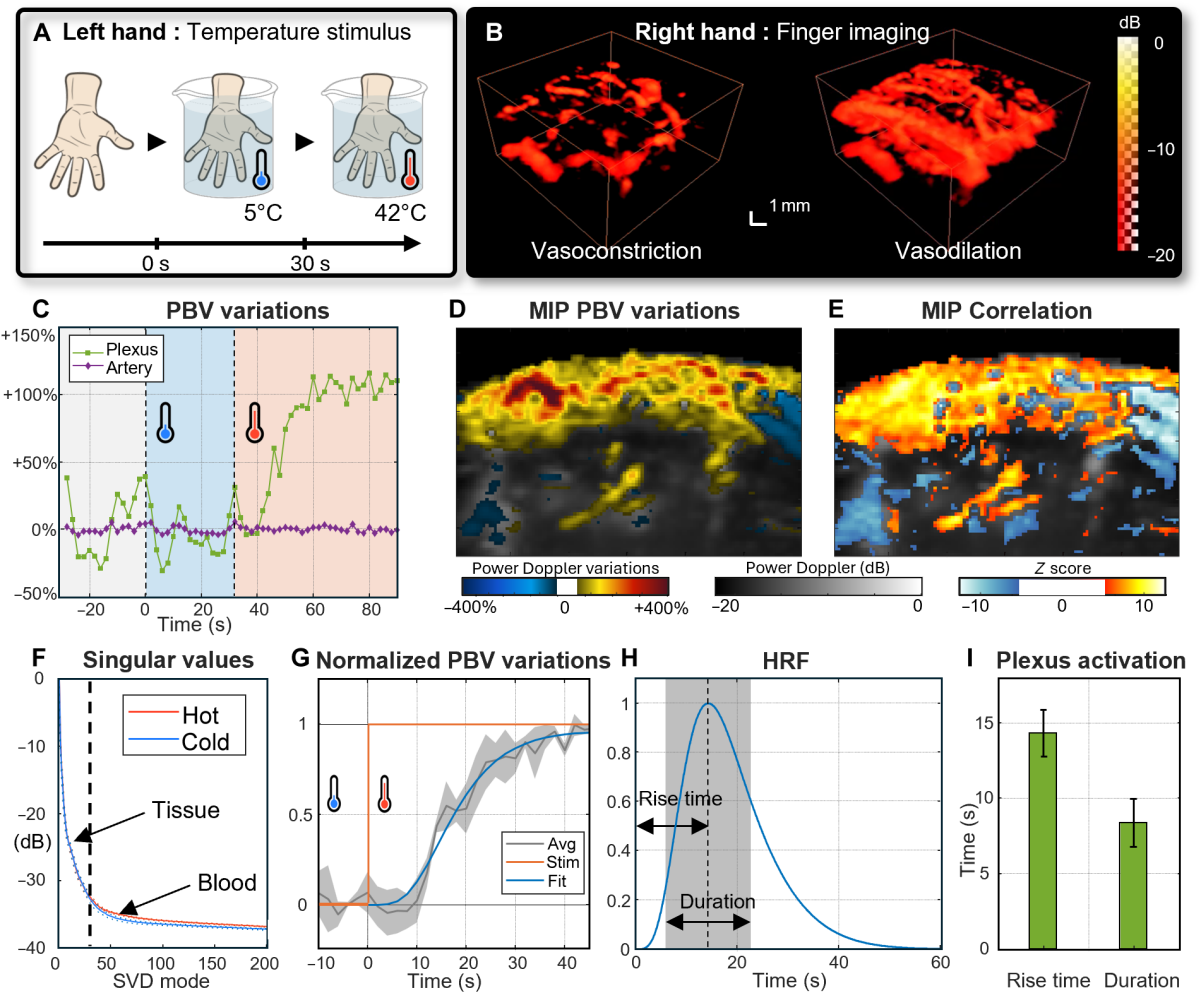
Systemic peripheral vasomotion monitoring. (**A**) Schematic of the thermal stimulus on the left hand (**B**) while power Doppler imaging is performed on the ring finger of the right hand to monitor systemic vasomotion reflex (movie S4). (**C**) Peripheral blood volume (PBV) variation monitored by power Doppler variations in the subcutaneous plexus and in the deeper artery. In the plexus, the cold stimulus provokes a sudden PBV decrease, but the average level does not change significantly in comparison with the baseline. Then, the hot stimulus significantly increases the PBV (+113 ± 7%) in comparison with the baseline (Welch test, *P* = 1.5 × 10^−6^). (**D**) Signed maximum intensity projection (MIP) of the blood volume variation in the finger on top of the power Doppler. Variations up to +577% in the superficial layers of the plexus. (**E**) Slice of the *z*-score computed with the Fisher *z*-transformation of the correlation between the PBV variations and the thermal stimulus. The variations are correlated with the stimulus in the plexus (|*z*| > 5.4 corresponds to *P* < 0.001 for a multi-one-tailed test, considered as significant). (**F**) Average and SD (dotted line) of the singular values of the SVD modes for hot acquisition and cold one. Only blood modes change. (**G**) Averaged normalized PBV variation (four acquisitions) in the subcutaneous plexus fitted with a cumulative difference of gamma distribution and the Heaviside thermal stimulation. (**H**) Hemodynamic response function (HRF) and the difference of gamma distributions of the fit. Rise time is measured between the stimulus and the maximum of the distribution, and duration is the SD of the distribution. (**I**) Comparison of the rise time and the duration of the vasomotion.

We used the systemic thermoregulation reflex to induce vasomotion as described in the schematic Fig. 5A. Hot stimulus on a highly vascularized peripheral area of the body (e.g., a hand) leads to a systemic vasodilation of the peripheral vascular system (e.g., the contralateral hand) and cold stimulus leads to a systemic vasodilation of the peripheral vascular system (e.g., the contralateral hand) to a systemic vasoconstriction. We selected the temperature and the stimuli length to induce a thermal body response without pain for the volunteer. This protocol on the left hand successfully induced vasomotion captured in the right hand with the CMUT RCA sensor power Doppler imaging (Fig. 5B). During exposure to 5°C, blood volume in the finger decreased and then increased above baseline during exposure to 42°C, before reaching a plateau. To quantify the phenomenon, we computed the variation of the power Doppler to get the peripheral blood volume (PBV). In the plexus, we measure a sudden short decrease of the PBV after the cold stimuli, but the mean level does not change significantly because of oscillations in the baseline. After the hot stimulus, the PBV increased strongly. We measured a significant variation of 113 ± 7% between the plateau reached and the baseline (Welch test, *P* = 1.5 × 10^−6^) (Fig. 5C). The activation detected is the strongest in the subcutaneous tissue (up to 577%) and in the vertical vessels that connect it to deeper arteries (Fig. 5D). We also calculated the *z*-score map that confirms a significant correlation between the stimulus and the power Doppler increase in the plexus (*z*-score = 5.4 corresponds to *P* = 0.001 for multiple one-way tailed tests) (Fig. 5E). Significant local anticorrelations are also noticeable, even if their amplitude is lower than the overall PBV increase. This results in a more complex activation pattern. Deeper tissues also show PBV variation, but no clear correlation could be measured. There is no variation in the singular values of the tissue modes (*1*–*30*) between vasodilation and vasoconstriction, which could be linked to a movement artifact. However, blood modes (bigger than 30) values are increased with vasodilation (Fig. 5F).

We also evaluated the activation delay in the different ROI and its reproducibility. We performed the cold/hot transition of the opposite hand on a healthy volunteer on four different days (*n* = 4) (Fig. 5G). Furthermore, we fitted the PBV signal with a difference of gamma (Fig. 5H) cumulative distribution and measured both the activation rise time and duration. A rise time of 14.3 ± 1.5 s and a duration time of 8.4 ± 1.6 s were measured in the plexus (Fig. 5I).

## DISCUSSION

Our study addresses a critical gap in eHealth sensor technologies by focusing on the monitoring of microvascular health. While existing wearable devices, such as smartwatches, excel at monitoring vital signs like the heart rate, ECGs, and oxygen levels, they fall short in providing comprehensive monitoring of micro vascularization.

We thus designed a 3D ultrasound method for microvascular monitoring using a CMUT RCA sensor, enabling high-resolution 3D Doppler imaging of microvascular structures. The sensor and sequences were optimized for imaging and low acoustic output, finding that the OPW with 32+32 plane-wave imaging provided the best results in resolution and contrast in Doppler flow phantoms, achieving a Doppler CNR of 19 dB in the volume.

In vivo, the sensor was able to image 3D microvascular flows in the fingers, wrists, and toes of volunteers, demonstrating consistent microvascular imaging over several months. Those results are in good agreement with 2D microvascular ultrasound imaging (*50*) and other imaging techniques such as PA (*51*, *52*) and OCT (*15*). Automatic 3D registration of the microvascular volumes significantly increased the correlation between power Doppler volumes from 49.0 to 74.9 on the same finger, with a minimum common volume of 84% of the sensor’s FOV. This proves reliable software-assisted repositioning can be achieved over 6 months. The sensor’s 3D capability is crucial for operator-independent monitoring, allowing for easier repositioning without the skill and training needed for 2D ultrasound imaging solutions. This feature is particularly valuable for integration into wearable devices, such as smartwatches or armbands, allowing users to use the device without complex training. The ability to accurately reposition the FOV between measurements ensures consistent data quality and reliability over long-term monitoring periods. Using microvascular volumes obtained from different fingers yielded a consistent lower correlation, suggesting the potential use of this volume as a vascular fingerprint for biometric identification.

The sensor effectively mapped microvascular hemodynamic changes in response to contralateral temperature variations, monitoring systemic vasomotion and demonstrating sensitivity to physiological flow variations. During the systemic thermoregulation reflex, blood volume in the imaged finger decreased by 27% during cold exposure (5°C) and increased by 64% during hot exposure (42°C) relative to baseline. The subcutaneous vascular plexus showed the highest correlation with temperature change stimuli, confirming its significant role in thermoregulation. Vasomotion during cold and hot exposure is in good agreement with 2D microvascular ultrasound imaging. These studies also show that extreme vasoconstriction level could be associated with scleroderma and complications including digital ulcers (*53*, *54*).

The sensor also assessed cardiovascular information on the basis of both on flow measurements in larger microvessels and from mechanical vibrations of the tissue. Spectral power Doppler could be formed in the plexus and was consistent with the one obtained with conventional ultrasound systems. Spectral Doppler imaging showed a clear pulsatile waveform that matches ECG data and could be used for RI computation. However, blood flow speed could not be measured due to the low angle of the vessels and aliasing was noticeable in bigger vessels. Using a Kasai estimator (*55*), tissue mechanical vibrations from ultrafast images allowed the detection of the pulsatility, heart rate, pulse time delay, and PWV when associated with a reference ECG and using a fixed heart-finger distance. The Bland-Altman plot of heart rate measured by the pulse vibration and ECG showed good accuracy (mean difference of 0.03) and precision (95% limits of agreement at ±1.7 for a mean value of 69.7). The delay between the ECG R-wave and the pulse wave arrival was measured at 214 ms with an SD of 8 ms, consistent with literature values (*3*). Compared to PPG measurements with a pulse oximeter on the same subject, the obtained waveform was much sharper and more closely matched the ECG-R waveform pointing to potential better accuracy to HRV and PWV measurements, two indicators of neurological and cardiovascular health. The amplitude and the frequency of the oscillations after the pulse wave peak could also be investigated as potential indicators for blood pressure and microvessel stiffness (*56*).

Previous works on PA imaging devices have demonstrated the capability to image the 3D structure of peripheral blood vessel systems, showing relationships between blood vessel structures and vascular age or health. For example, different teams (*18*, *51*) and Canon Inc. have shown that PA imaging can visualize vascular structures and relate them to health conditions. The CMUT RCA sensor achieves similar resolution in blood vessel imaging without requiring a laser. The vascular structures detected by the CMUT sensor closely match those imaged by PA devices, confirming the accuracy of the CMUT sensor in detecting subcutaneous plexuses. Microvascular PA devices often use higher frequency imaging than our CMUT RCA, for example, between 10 and 160 MHz for this study (*52*). The design of our sensor results in a trade-off between the FOV, the number of elements, and the pitch of the array. The sensor should not have more than 128 elements for electronic reasons, a wavelength-sized pitch to reduce grating lobes level, and, lastly, a 1-cm^2^ FOV to capture enough vascular information. With all these requirements, a 10-MHz frequency was an interesting choice. The vascular structures detected by the CMUT sensor closely match those imaged by PA devices, confirming the accuracy of the CMUT sensor in detecting subcutaneous plexuses with fewer channels and without requiring a laser. It also confirms the interest of our design. A comparison of the different imaging methods can be found in Table 3.

**Table 3.** Sequences tested in the phantom study.

Sequence	Emission	Plane waves ° (step °)	Element used/emission	Frames	Ultrasound frame rate (Hz)	Duration (s)
**SA**	64	–	1	400	312.5	1.28
**HSA**	64	–	64	400	312.5	1.28
**OPW**	32+32	−15.5:15.5 (1)	64	400	312.5	1.28
**OPW**	16+16	−7.5:7.5 (1)	64	800	625	1.28
**SPW**	64	−31.5:31.5 (1)	64	400	312.5	1.28
**SPW**	32	−15.5:15.5 (1)	64	800	625	1.28

LDF has also been extensively used to monitor hemodynamic variations in the skin and their role in thermoregulation. LDF studies, including those by (*57**–**59*), have shown the ability to evaluate different physiological processes through wavelet decomposition. The vasomotion time constants measured by the CMUT sensor align with the myogenic oscillation (0.05 to 0.15 Hz) driven by the sympathetic nervous system reported in LDF studies. While LDF has low hardware requirements, making it convenient, its limited depth penetration and lack of 3D imaging capabilities are significant drawbacks compared to the CMUT sensor. Studies using LDF have shown that various parameters, such as body temperature, exercise, stress, breathing, and caffeine intake, can affect limb perfusion. The CMUT sensor’s ability to simultaneously access heartbeat and vasomotion data could provide deeper insights into the trade-offs between heat dissipation and blood redirection during prolonged physical effort or intense emotional stress.

Despite these advancements, our study and prototype face several limitations. The use of water for acoustic coupling, while effective in a controlled experimental setup, is impractical for everyday use in wearable devices. Alternative coupling methods, such as hydrogel patches, have been proposed and could offer a more practical solution to enhance usability and practicality in real-world applications. Recent studies have shown the feasibility of using such hydrogel patches for ultrasound coupling for various imaging modes, including M-mode, 2D B-mode, 3D elastography, and PA (*19*, *25*, *26*). This kind of coupling could be useful for longitudinal studies during hours or even a few days, enabling better understanding of hemodynamics and help develop new quantitative biomarkers (*60*).

Movement artifacts remain a potential source of noise in the data, despite efforts to minimize them through custom 3D printed holders and stable coupling mediums (fig. S3). Studies have shown that motion artifacts can significantly affect the quality of Doppler imaging and functional analysis of hemodynamics, especially for smaller blood flows (*61*). Future designs should incorporate motion compensation algorithms such as dynamic thresholding (*62*), real-time motion artifacts detection and censoring, as well as hardware solutions to better secure the sensor against the skin. The influence of various pressure levels of the sensor on the skin could also create biases in the microvessel’s readouts by compressing the smaller vessels. Last, deeper musculoskeletal vessels might be missing in our acquisitions due to lower sensitivity in depth, for example, in the muscle fibers or in joints.

The acquisition was performed using an ultrasound-research platform (MODULEUS, Tours, France) in this study, which is not viable for wearable applications as it is primarily designed for ultrasound research. The sensor itself was attached to a custom rigid printed circuit board (PCB), which is not ideal for measurements in some parts of the body such as the neck or limb. The sensor power consumption and processing required remain relatively high in comparison to current fully wearable devices (*63*, *64*). Future research should focus on optimizing the sensor’s operating parameters, improving the efficiency of acquisition electronics, and leveraging custom ASIC chips. Recent advancements in portable ultrasound devices, such as the Butterfly IQ systems (*47*) or those by this team (*20*), demonstrate the potential for lightweight, battery-powered portable systems.

The study was also conducted on a limited number of healthy volunteers, which may not fully represent a diverse population or various health conditions. While the initial results are promising, the performance of the CMUT RCA sensor must be validated across a broader demographic spectrum, including individuals of different ages, genders, ethnic backgrounds, and those with various clinical conditions such as diabetes, hypertension, autoimmune diseases, and cardiovascular disorders. The impact of such disease progression on the microvasculature over several months would also be of major interest, as they could potentially serve as early biomarkers.

Integrating such repositionable 3D microvascular sensors into wearable devices could provide unprecedented insights into microvascular health. This would enable long-term monitoring and comparison of readings over months or years, facilitating research, early detection, and monitoring of disease progression at both the patient and population levels.

## MATERIALS AND METHODS

### Design and fabrication of the RCA CMUT transducer

The CMUT chips were designed by MODULEUS (Tours, France) for microvascular imaging applications, optimized to operate at a central frequency of 10 MHz with a pitch of 150 μm, corresponding to a wavelength in water. The wafer manufacturing of the CMUT chips followed standard semiconductor processes, such as thin-film deposition, photolithography, and etching to fabricate the flexible membranes and cavities on silicon substrates. Post-fabrication, the wafers were diced into individual chips.

Each CMUT cell is structured with flexible electrodes separated by a vacuum-sealed cavity, which collapses under a 35-V dc bias voltage, while a 15-V ac voltage is used to vibrate the membrane, generating ultrasound waves (Fig. 1D). These elements were then soldered onto custom-designed PCBs. The PCBs feature micro-fabricated connections and solder pads to ease electrical interfacing. A passivation layer, ~200 μm thick, was applied to the CMUT surface to provide mechanical and chemical protection. This protective layer ensures the robustness of the sensor against environmental factors during usage.

The assembled CMUT RCA sensor incorporates 64 rows and 64 columns, using row-column addressing to reduce the number of required channels, thus simplifying the electronic complexity and reducing costs. To minimize signal loss and keep high data fidelity, the CMUT arrays were directly attached to the PCBs using flip-chip bonding. This design also provides reliable electrical and mechanical connections.

### Transducer characterization

The acoustic characterization of the CMUT RCA sensor was conducted using pulse-echo measurements on a water/air interface. A water tank was constructed right atop the PCB to provide a proper acoustic coupling medium. The sensor was accurately aligned to ensure accurate measurements. Each sensor element, including all rows and columns, was evaluated individually using a MODULEUS ultrasound platform.

During the acquisition, a 35-V dc bias voltage was applied along with a ±15-V ac excitation voltage to collapse the cavity and generate ultrasound waves. The ultrasound signals received were sampled at a rate of 100 MHz by the CMUT RCA sensor. The data collected from these pulse-echo measurements were used to compute the frequency spectrum of the signals received to determine the sensor’s center frequency and bandwidth.

Impedance measurements were performed using a 4294A Precision Impedance Analyzer (Agilent Technologies, Santa Clara, CA, USA) to verify the electrical characteristics of the sensor elements. The power consumption of the sensor was simulated on the software LTspice with these parameters.

### Phantoms fabrication

To evaluate the CMUT RCA sensor’s imaging capabilities, two types of phantoms were fabricated: a bead phantom and a flow phantom. The bead phantom was constructed using PVC (sound speed 1480 m/s) to simulate tissue properties. A 180-μm–diameter steel bead was embedded at a depth of 6 mm within the PVC material. The construction involved pouring liquid PVC into a mold, positioning the steel bead at the desired depth, and allowing the PVC to solidify. This phantom was used to assess the sensor’s spatial resolution by providing a point target for PSF measurements.

The flow phantom was designed to simulate blood vessels and evaluate the sensor’s Doppler imaging performance for the different sequences. The phantom was made using a mixture of 7% gelatin, 1% agar, and 5% propan-1-ol dissolved in water to mimic the acoustic properties of soft tissue. The gel mixture was degassed using an A-1000S vacuum pump (Eyela World, New York City, NY, USA) to remove air bubbles and then heated to 60°C before being poured into a mold containing a 500-μm–diameter wire. After the gel solidified, the wire was removed to create a cylindrical cavity simulating a blood vessel.

For the flow measurements, the cavity was connected to a syringe pump (KDS 100, KD Scientific Inc., Holliston, MA, USA), which circulated a blood-mimicking fluid (Shelley Medical Imaging Technologies, London, ON, Canada). The fluid was composed of water, glycerol, and a small number of scattering particles to mimic the acoustic properties and flow characteristics of blood. The flow rate was controlled at 1 cm/s to provide a consistent and reproducible flow within the phantom.

### Sequences

Different sequences were investigated to optimize the power Doppler imaging on the CMUT RCA sensor. These methods are based on plane waves and single-element SA imaging. They are described in Table 3.

The sequences were first simulated using the Field II software (*65*, *66*) for a first screening of the imaging parameters. They were then implemented on a fully programmable research scanner from MODULEUS (Genesis, MODULEUS, Tours, France) for both ac and dc. Control of the ultrasonic scanner and all postprocessing were conducted using MATLAB R2021a (MathWorks, Natick, MA, USA).

SA: Each element transmits sequentially, and only one array is used for emission due to the number of required transmits. This method is useful for high-resolution imaging (*48*).

HSA imaging: Elements transmit positive or negative polarity pulses with a Hadamard-based pattern. Linear operations between the different events re-form the SA received signal with a higher SNR. This method requires sensor symmetry to ensure accurate signal reconstruction and improved image quality (*49*).

SPW: Plane waves are transmitted by applying a linear delay law between each of the transducers. This method involves the emission of plane waves on only one array, followed by compounded frames of different numbers of plane waves to assess system resolution.

OPW: As described by in this study (*67*), this method involves the emission of plane waves with the two arrays sequentially, with cylindrical focusing in transmission along rows and orthogonal cylindrical focusing in reception along columns, and vice versa. This approach increases the symmetry of the PSF by combining the sequences of column reception and row emission and row reception and column emission. The transmitted beams fit the geometry of the RCA probe, with angles set to avoid grating lobes (Δθ = 1°).

For OPW acquisitions, XDoppler was also performed. Instead of computing a coherent summation of the volumes obtained with columns and rows, the volumes were correlated, following the method described here (*68*).

### Acoustic output measurements

Safety evaluations were conducted to ensure the CMUT RCA sensor’s compliance with regulatory standards. A hydrophone (HGL-0085, Onda Inc., Sunnyvale, CA, USA) was used to measure the acoustic output of the CMUT RCA sensor. The hydrophone was mounted on a three-axis motorized stage to enable precise scanning in the *X*, *Y*, and *Z* directions within a water tank constructed atop the sensor. The sensor’s output was driven by a MODULEUS ultrasound scanner.

During the measurements, a 10-MHz, two-cycle plane wave with a theta of 0.1° was transmitted into the water. The hydrophone detected the resulting pressure waves, and the data were used to calculate key safety metrics according to AIUM/NEMA guidelines. Specifically, the MI, ISPTA, and thermal index were computed according to the different sequences used.

### In vitro measurements

For the spatial resolution measurements, a custom PVC phantom containing a 180-μm–diameter metal bead was used. The phantom was set on top of the CMUT RCA sensor and coupled with ultrasound gel. B-mode imaging of the metal bead was performed to capture the PSF profiles. The acquired data were beamformed to reconstruct the 3D volume. The FWHM was then computed from the PSF profiles to determine the spatial resolution of the imaging system. These measurements were repeated for each imaging sequence to evaluate and compare their performance.

For the flow CNR measurement, the flow phantom was used. Power Doppler imaging was performed to acquire 3D flow data. The CNR was computed by dividing the mean signal intensity within the vessel by the SD of the background noise outside the vessel. This procedure was repeated for different imaging sequences to assess and compare their flow detection sensitivity.

### In vivo Doppler measurements

In vivo Doppler measurements were conducted to evaluate the performance of the CMUT RCA sensor in detecting microvascular changes in human subjects at several locations: fingers, wrist, and toe. The study involved three healthy volunteers who provided informed consent before participation.

The setups for the in vivo measurements were fabricated using custom 3D printed holders designed to maintain consistent positioning relative to the target body part. The holders were equipped with Velcro straps to minimize movement artifacts during imaging. Each holder consisted of a small, closed tank with a 13 mm–by–13 mm window on top. The finger, toe, or wrist was placed in the opening, and the 2-mm spacing between the probe and the skin was filled with degassed water to ensure stable acoustic coupling. A stop wall limited movement in the longitudinal direction, while the strap band in the vertical direction. It was gently tightened to avoid vein compression in the imaging area.

The imaging sequence used for the in vivo power Doppler measurements was the OPW sequence with 32+32 transmits at a pulse repetition frequency (PRF) of 20 kHz for 200 or 400 frames and acquisition durations of 0.64 or 1.28 s, respectively, or a PRF of 3 kHz for a duration of 8.5 s.

After 3D beamforming, a mask was automatically applied to the volume outside the finger, toe, or wrist to remove potential microbubbles on the skin. The depth of the finger was obtained by fitting the B-mode image with a polynomial surface, and the volume above this surface was set to zero. The microbubbles create minor hyper signals that may slightly interfere with the clutter filtering and reduce the power Doppler image quality. However, they do not significantly affect the overall acoustic signal from the finger.

Clutter filtering was then performed using SVD, which separated the tissue signal, blood flow signals, and electronic noise. For Doppler imaging, the first 15% of the modes and the last 50 modes, considered mostly as tissue signal and electronic noise, were removed.

### In vivo longitudinal Doppler measurements

Longitudinal Doppler imaging was performed to evaluate the capability of slow-time monitoring of microvascular volumes. Repeated Doppler measurements were taken from the same ring fingers at various times, days, and months. Acquisition on the index finger was also added for comparison. This approach aimed to assess the ability to image the same volume with rough repositioning over time using a consistent setup.

To further improve the alignment of the volumes, a rigid registration of the subcutaneous plexus vessels was performed. The vessels were first segmented using vesselness filters based on Hessian matrix (3D implementation on MathWorks file exchange, https://github.com/timjerman/JermanEnhancementFilter) (*69*) and a threshold on the 3D power Doppler volumes. Then, a rigid registration process (function Imregister, MathWorks, MATLAB) aimed to refine the spatial alignment of the Doppler images between imaging sessions. To ensure the reliability of the imaging system in capturing consistent microvascular volumes over extended periods, correlation analysis between the acquired volumes with or without registration was conducted.

### In vivo pulsatility measurements

Pulse wave measurements were conducted to evaluate the CMUT RCA sensor’s capability in capturing cardiovascular pulsatility in the finger. Frame-to-frame correlation was performed to image the pulse wave in the finger. The Kasai phase shift estimator (*55*) was computed on the frames before clutter filtering. This phase shift is an estimator of the *z*-axis displacement between consecutive frames, allowing for the detection and quantification of tissue motion. The pulse wave induces localized tissue contractions around the deepest arteries: the MPW, while finger movements result in global artifacts (rotation or translation).

To remove artifacts, SVD was performed on the Kasai volumes, retaining the first five modes to describe the different movements of the finger during acquisition (*70*). Because SVD decomposition is not specific to pulse wave imaging, it mixes different movement causes. Independent component analysis was then used on the temporal vectors to separate the different movement sources (pulse wave and artifacts). Features describing the pulse wave were identified through their power spectral density.

Three ECG electrodes (MSGLT-09 Solid, Medico LTD, Noida, Uttar Pradesh, India) were applied to the left and right wrists, as well as to the left hip linked to an ECG device (AccuSync 72, AccuSync Medical Research Corporation, Milford, CT, USA) that automatically detected the R-wave of each heartbeat and was used to trigger ultrasound scanner to synchronize its transmissions with the heartbeat.

The arrival time of the pulse wave was compared to the ECG. PWTT was defined as the time difference between the R-peak and the maximum of the MPW deformation. The PWV was then calculated by dividing the measured distance between the heart and the finger of the volunteer (88 cm) by this PWTT.

To compare the pulse wave shape with the one obtained from plethysmography, a pulse oximeter (CMS 60D, Contec Medical Systems Co., Qinhuangdao, China) was used, and the data were extracted and realigned.

### In vivo spectral Doppler measurements

Spectral Doppler measurements were computed on the finger blood vessels using a continuous 10-s acquisition of the OPW8+8 to increase the frame rate to 1250 frames/s and limit aliasing. A 30-point window short-time Fourier transform was applied to the temporal blood signal of the segmented blood vessels. The resulting spectrum is an estimator of the blood speed along the *z* axis. The spectral Doppler displayed is the average of the spectral signal in the ROI of 3 × 3 × 3 voxels underlined in the signed power Doppler volume.

The RI, a common vascular resistance metric, was then computed. This index is independent of the imaging angle and does not require angular correction. It is calculated using the method described in this study (*71*).

For comparison, acquisitions have been done with a clinical ultrasound scanner (Aixplorer, SuperSonic Imagine, Aix-en-Provence, France) and a 15-MHz linear probe. Signed power Doppler and spectral Doppler of plexus vessels have been computed and displayed. The RI has been computed as an internal feature of the ultrasound scanner on the spectral Doppler acquired during four cardiac cycles. Fifteen different acquisitions have been made on different vessels of the subcutaneous plexus by removing and replacing the probe.

### In vivo hemodynamic variations during thermoregulation

The study on in vivo hemodynamic variations during thermoregulation involved imagining the right hand of a healthy volunteer, while the left hand was subjected to different thermal conditions. Specifically, the left hand was immersed in either 5° or 42°C water to induce thermoregulation vasomotion reflexes: PBV decreases during cold exposure and increases during hot exposure.

To capture these systemic hemodynamic variations, the following protocol was used. The right hand is imaged with a power Doppler image frequency of 0.5 Hz for 2 min. The baseline was acquired at room temperature (22°C) for the first 30 s. Then, the left hand of the volunteer was immersed in 5°C water during the next 30 s. Last, the left hand was immersed in 42°C water during the remaining 60 s.

The data were analyzed to quantify the blood volume changes and assess the responsiveness of the subcutaneous vascular plexus. The mean power Doppler variation in two areas (the plexus and the deeper artery) was computed to assess the global power Doppler variations. Then, the changes in each pixel were calculated, and correlation with the temperature stimuli was computed with the Fisher *z*-transformation. Bonferroni correction was applied to correlation maps to consider the multiple tests. The number of tests was set to the imaging volume divided by the volume of the PSF.

The hemodynamic response was fitted with a difference of gamma distribution convoluted with a Heaviside function because of the length of the stimulus. Time metrics are then computed on this distribution. The rise time is the time between the stimulus and the maximum of the distribution, and the duration is the SD of the distribution.

### Statistical analysis

All results of the article are reported as means ± SD. We assessed the normality of our sample distribution with the Shapiro-Wilk normality test. Because the test was positive, we assumed the normality of the data. To compare paired samples, before and after the registration, we used the paired *t* test. To compare different independent samples for method comparison, we used the Welch test. The significance thresholds used were ***P < 0.01 and ****P < 0.001. Last, we used Bonferroni corrections for the *z*-score maps because of the high number of tests.
