# Supplementary material for: Monitoring microvascular changes over time with a repositionable 3D ultrasonic capacitive micromachined row-column sensor
Source: Sci Adv. 2025 Mar 26;11(13):eadr6449. doi: 10.1126/sciadv.adr6449 (PMC11939045; doi:10.1126/sciadv.adr6449)
Supplement: Supplementary file 1 — Figs. S1 to S4 Legends for movies S1 to S4 [file sciadv.adr6449_sm.pdf]

Science Advances
AAAS

Supplementary Materials for

Monitoring microvascular changes over time with a repositionable 3D ultrasonic capacitive micromachined row-column sensor

Cyprien Blanquart *et al.*

Corresponding author: Thomas Deffieux, thomas.deffieux@inserm.fr

Sci. Adv. **11**, eadr6449 (2025)
DOI: 10.1126/sciadv.adr6449

The PDF file includes:

Figs. S1 to S4
Legends for movies S1 to S4

Other Supplementary Material for this manuscript includes the following:

Movies S1 to S4

- **Movie 4. Vasodilation**

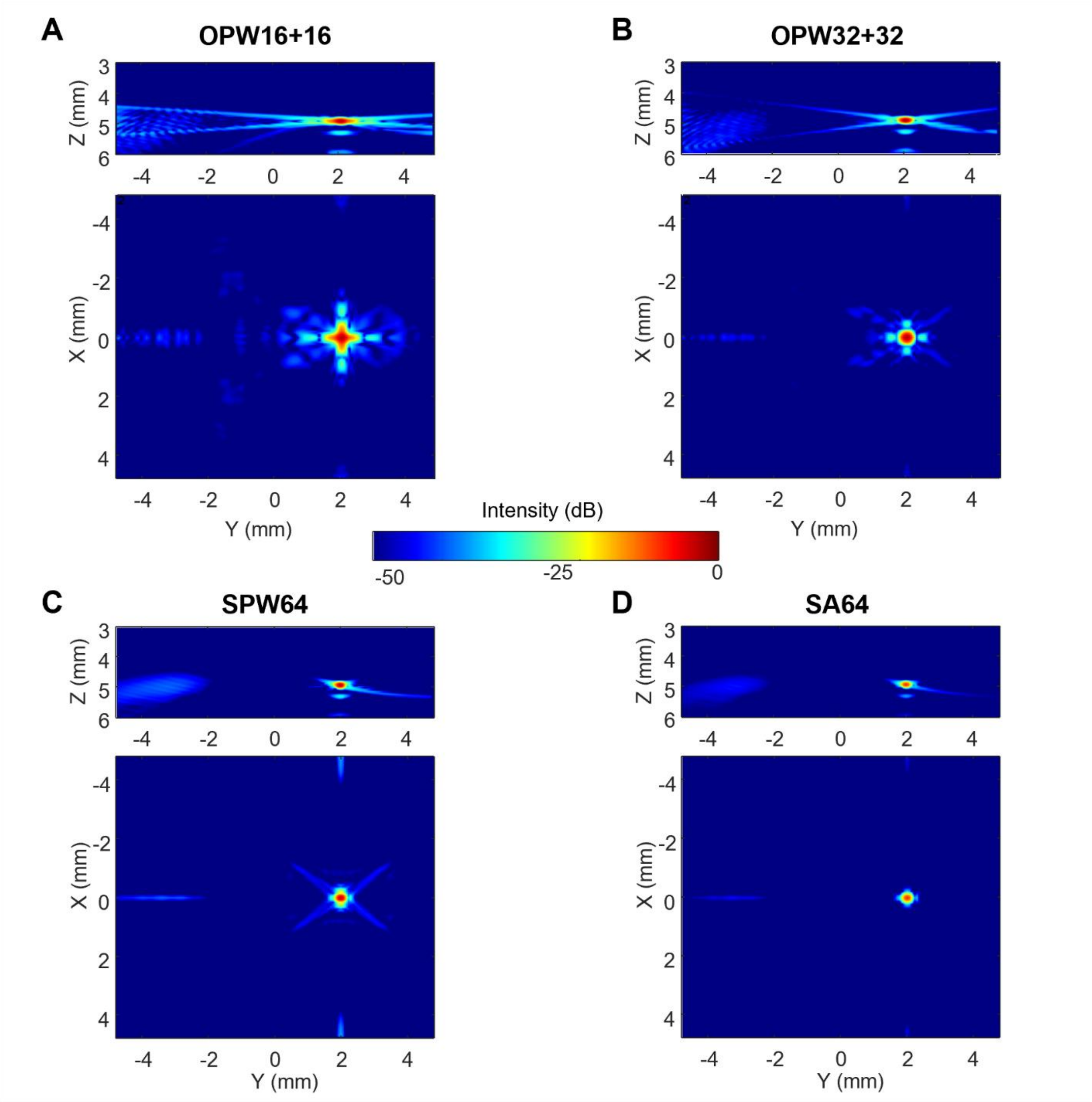

Fig. S1. Simulated Point Spread Function

XY and ZY slices of simulated PSF for different RCA imaging sequences. The simulated data are computed with FieldII with a scatterer in (X = 0 mm, Y = 2 mm, Z = 5 mm). **A**) OPW16+16, **B**) OPW32+32, **C**) SPW64 and **D**) SA64. The PSF shows sides lobes due to the plane wave or synthetic aperture focalization. Grating lobes are due to probe pitch.

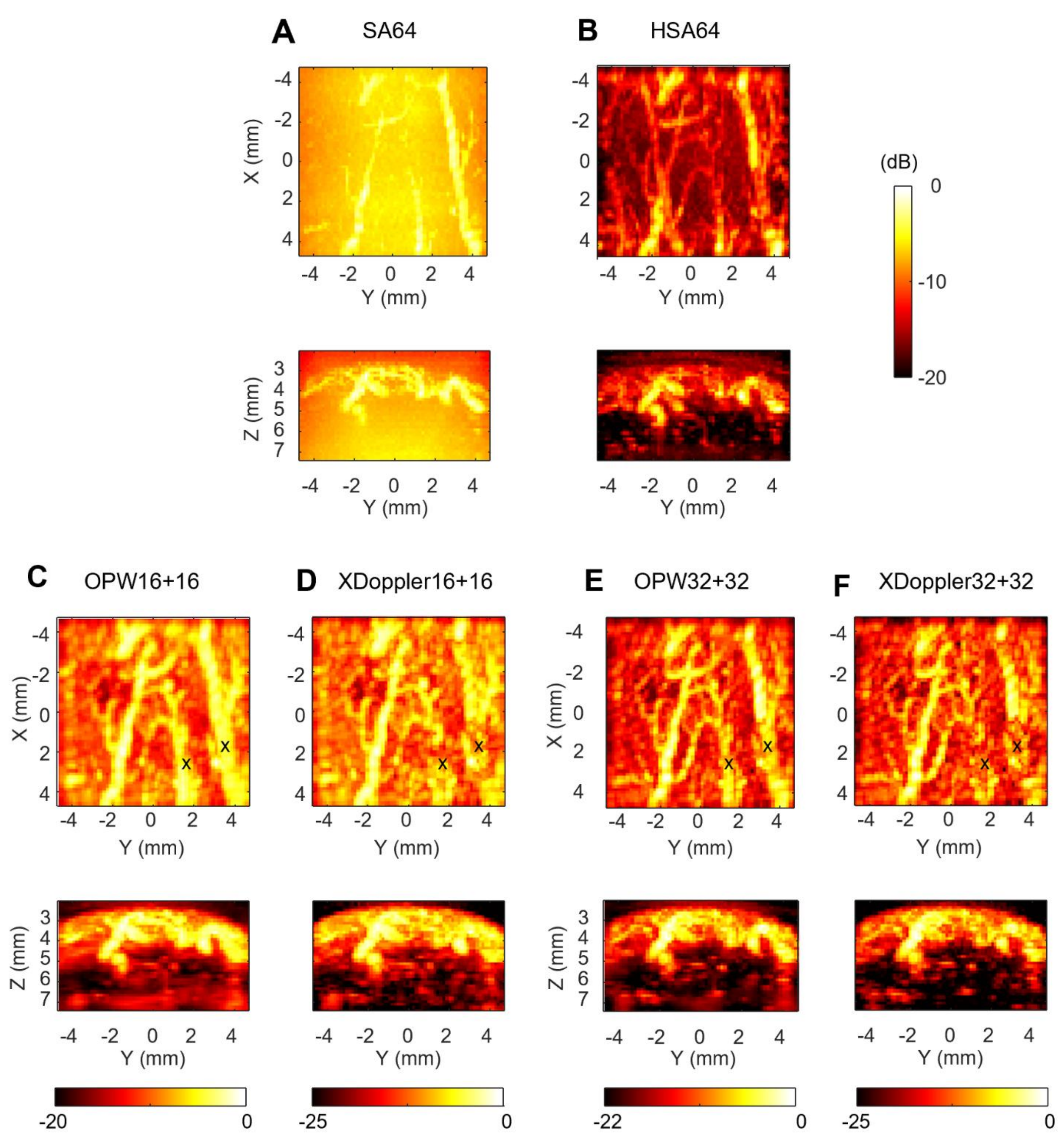

Fig. S2. *In vivo* comparison of the different sequences

Comparison of the power Doppler of the ring finger (MIP) with different imaging sequences. Acquisitions are made in a row to limit physiological variations. The PRF is 3 kHz, and the acquisition duration is kept at 8.5 s for all the acquisitions. OPW32+32 and XDoppler32+32 are obtained with different processing of the same acquisitions. **A**) SA64 (400 frames), **B**) HSA64 (400 frames), **C**) OPW16+16 (800 frames), **D**) XDoppler16+16 (800 frames), **E**) OPW32+32 (400 frames) and **F)** XDoppler32+32 (400 frames).

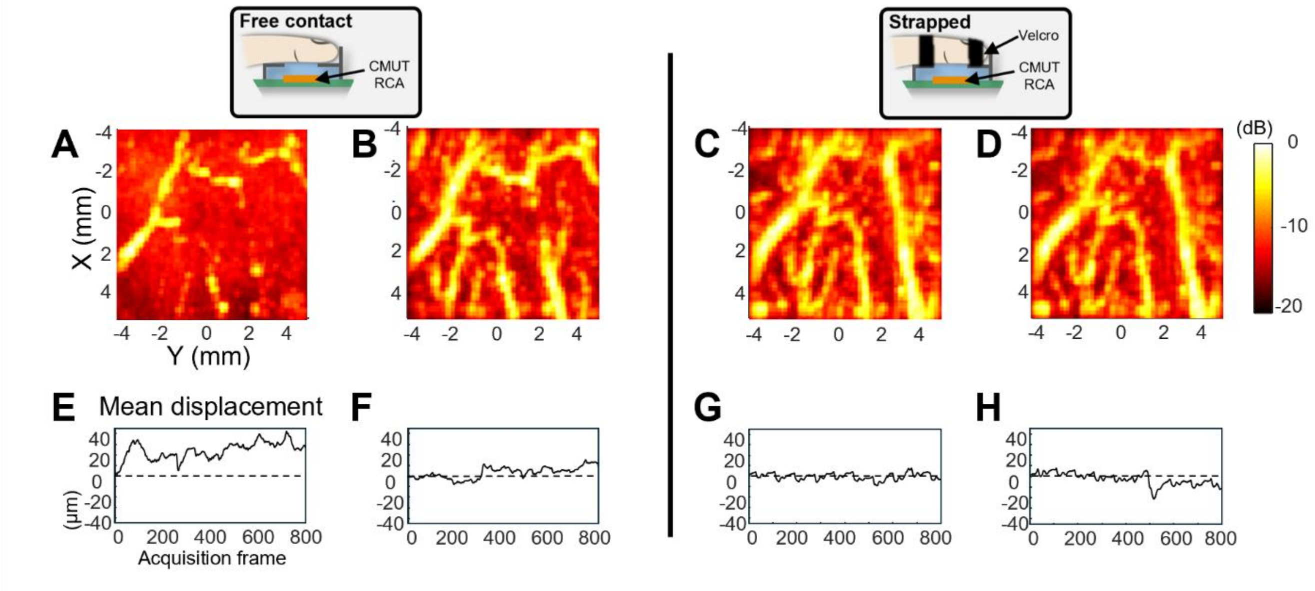

Fig. S3. Movement during the acquisition

A, B, C, D) Projection power Doppler of the ring finger of a volunteer with a free contact finger support **(A, B)** and with the strapped finger support we used in this study **(C, D)** (sequence: 8.5 s, PRF: 3 kHz, sequence OPW16+16). Even while trying to stay still, movement can appear in case of free contact, reducing the image quality as seen in (**A**). **E, F, G, H)** Global movements of the finger are tracked thanks to Kasai frame-to-frame lag autocorrelation, averaged on the volume for the different acquisitions.

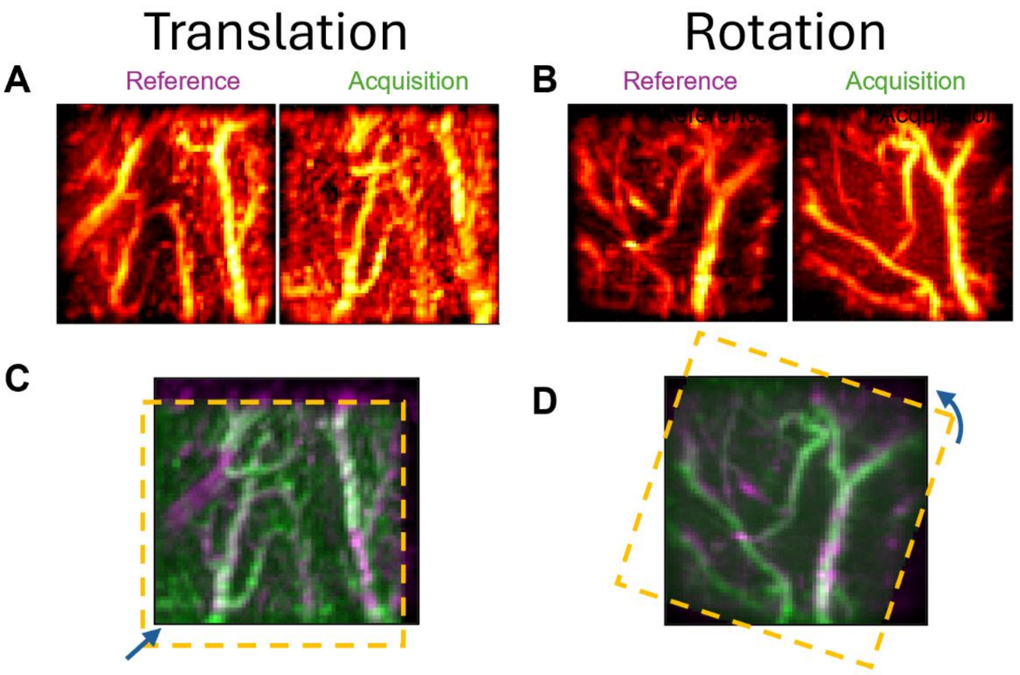

Fig. S4. Reposition between the acquisitions
Example of the reposition of 2 acquisitions with different fields of view in the case of translation (OPW32+32) **(A)** and rotation (OPW16+16) **(B)**. The merged volumes are displayed with two colors (reference: purple, new acquisition: green) **(C, D)**.

Movie 1. 3D power Doppler 1
Power Doppler of a finger (OPW32+32, 400 frames, PRF 20 kHz), in rotation to display the plexus pattern.

Movie 2. 3D power Doppler 2
Power Doppler of the same finger (OPW32+32, 400 frames, PRF 20 kHz), in rotation to display the plexus and deep artery.

Movie 3. Pulse wave in the finger
Pulse wave displayed as a phase shift in the tissue around the artery displayed with the power Doppler of the same finger (OPW32+32, 400 frames, PRF 20 kHz).

Movie 4. Vasodilation
Power Doppler movie showing the vasodilation of vascular network of the finger during contralateral hot stimulus.
